# Emotion Regulation in Essential Hypertension: Roles of Anxiety, Stress, and the Pulvinar

**DOI:** 10.3389/fnbeh.2020.00080

**Published:** 2020-05-28

**Authors:** Avigail Wiener, Christiane S. Rohr, Navot Naor, Arno Villringer, Hadas Okon-Singer

**Affiliations:** ^1^Department of Psychology, School of Psychological Sciences, University of Haifa, Haifa, Israel; ^2^The Integrated Brain and Behavior Research Center (IBBR), University of Haifa, Haifa, Israel; ^3^Hotchkiss Brain Institute, The University of Calgary, Calgary, AB, Canada; ^4^Mathison Centre for Mental Health Research and Education, The University of Calgary, Calgary, AB, Canada; ^5^Department of Radiology, Cumming School of Medicine, The University of Calgary, Calgary, AB, Canada; ^6^Department of Psychology,University of Maryland, College Park, MD, United States; ^7^Max Planck Institute for Human Cognitive and Brain Sciences, Leipzig, Germany; ^8^Department of Cognitive Neurology, University Hospital Leipzig, Leipzig, Germany

**Keywords:** essential hypertension, emotion regulation, anxiety, stress, pulvinar

Excessive emotional arousal can impair individuals' ability to function and achieve their goals. This is especially true when this heightened arousal emerges from an emotional stimulus that is irrelevant to current goals and hence should be ignored (Ochsner et al., 2012). One clinical population that has yet to be investigated in the context of emotion regulation comprises patients with essential hypertension (EH). EH is defined as systolic blood pressure (BP) higher than 140 mmHg and/or diastolic BP higher than 90 mmHg (James et al., 2014). EH is the most important risk factor for cerebrovascular diseases, a major cause of death in industrialized societies (Mendis et al., 2011; Mozaffarian et al., 2016). Frequent complications of EH include atherosclerotic coronary artery disease, congestive heart failure, stroke, Alzheimer's disease and chronic kidney disease, and therefore constitutes a leading cause of severe disability and premature death (Mendis et al., 2011; James et al., 2014; Mozaffarian et al., 2016).

Patients with EH exhibit “exaggerated” reactions to emotional and stressful stimuli (Jern et al., 1995; Deter et al., 2007), as well as high levels of anxiety (Liu et al., 2017). Recent evidence further suggests that patients with EH exhibit altered structure, function and connectivity within a neural network that has been associated with emotion regulation, which includes prefrontal and limbic regions (defined as the amygdala, insula, and cingulate cortex; Gianaros and Sheu, 2009; Jennings and Zanstra, 2009). Taken together, these different lines of investigation suggest possible abnormalities among patients with EH in neurocognitive inhibitory dysfunction, as related to emotion regulation, depression, anxiety, stress regulation, and emotion control processes. Yet to date very little research has examined possible deficits in cognitive control mechanisms, which may be the basis for the aforementioned emotion-related abnormalities in EH.

## Deficient Emotional Behavior in Essential Hypertension

Research has established that the tendency to exhibit enhanced cardiovascular responses to stress and aversive situations predicts later development of EH (Matthews et al., 2004; Gianaros and Sheu, 2009; Gianaros et al., 2012). Such responses include BP elevations that are higher than what is required for adaptive motor reaction to possible stressors (Lang et al., 1998, 2000). Researchers have posited that these “exaggerated” cardiovascular responses may be caused by abnormal neural circuits related to vascular control and reactivity to stress, eventually influencing the brainstem nuclei that control autonomic nerve movement to the myocardium and vasculature (Gianaros and Sheu, 2009; Gianaros et al., 2012). Patients with EH exhibit structural and functional abnormalities in neural networks that include fronto-parietal, limbic, and brainstem regions (Gianaros and Sheu, 2009; Gianaros et al., 2009; Jennings and Zanstra, 2009). Initial studies among healthy individuals demonstrate a relation between enhanced BP reactions and enhanced neural activation in limbic and brainstem regions in response to mental stress (Gianaros and Sheu, 2009; Gianaros et al., 2012). Based on these studies, researchers have suggested that brain abnormality in groups at high risk of developing EH is related to exaggerated BP responses to stress, which may play a *causal* role in the development of EH (Jennings and Zanstra, 2009). They speculate that such recurring “exaggerated” cardiovascular responses may promote structural changes in the vascular tissues and thus ultimately lead to the development of EH (Gianaros and Sheu, 2009).

## Enhanced Anxiety and Depression in Essential Hypertension

The association between chronic stress and EH is well-established (Lucini et al., 2005; Huang et al., 2013). Epidemiological studies have found that the association between anxiety and EH is bidirectional, such that individuals with EH are more likely to have anxiety and vice versa (Ginty et al., 2013; Liu et al., 2017).

There is also evidence for a relation between depression and EH (Davidson et al., 2000; Ginty et al., 2013). Depression is associated with changes in the autonomic regulation of the heart that are also associated with EH (Grippo and Johnson, 2009). In addition, depressive symptoms are related to inflammatory factors (Howren et al., 2009) that may affect the development of EH (Montecucco et al., 2011). Accordingly, integrated treatment for depression and EH has led to lower BP as well as fewer depressive symptoms, compared to usual EH treatment (Bogner et al., 2013; McClintock and Bogner, 2017). Nevertheless, observations of EH's association with anxiety and depression are inconsistent (Cheung et al., 2005; Hildrum et al., 2011; Wiltink et al., 2011). It is therefore crucial to further investigate and shed light on the underlying mechanisms.

## Are Abnormal Emotional Reactions in Essential Hypertension Mediated by Dysfunctional Neurocognitive Inhibition Mechanisms?

Studies with clinical and sub-clinical populations exhibiting anxiety and depression symptoms point to deficits in inhibition and control systems. Dysfunctional inhibitory mechanisms have been suggested as underlying cognitive control deficits in depression (Goeleven et al., 2006; Owens et al., 2013) and anxiety (Berggren and Derakshan, 2014). Correspondingly, abnormalities in prefrontal-limbic neural pathways have been shown both in depression (Drevets, 2000) and in anxiety (Bishop, 2008). For example, frontal and limbic activation during implementation of cognitive inhibition (manipulated by a Go/No-Go task) predicted post-treatment improvement of depression symptoms (Langenecker et al., 2007).

Do similar neurocognitive inhibitory dysfunctions mediate abnormally enhanced BP reactions? To date, most research examining the mechanisms responsible for EH has focused on the peripheral nervous system and peripheral BP (Jennings and Zanstra, 2009). Yet recent studies point to deficiencies in central regulatory factors such as central control of baroreceptor function and regulation mechanisms within midbrain areas (Gianaros and Sheu, 2009; Jennings and Zanstra, 2009; Gianaros et al., 2012). In addition, brain abnormalities such as altered cerebral blood flow, white matter hyperintensities, decreased gray matter volume, and brain atrophy are also associated with EH (for review, see Jennings and Zanstra, 2009). Additional evidence shows that EH is related to cognitive impairment, deficits in executive function and processing speed as well as dementia (Hughes and Sink, 2016).

**Figure 1:**
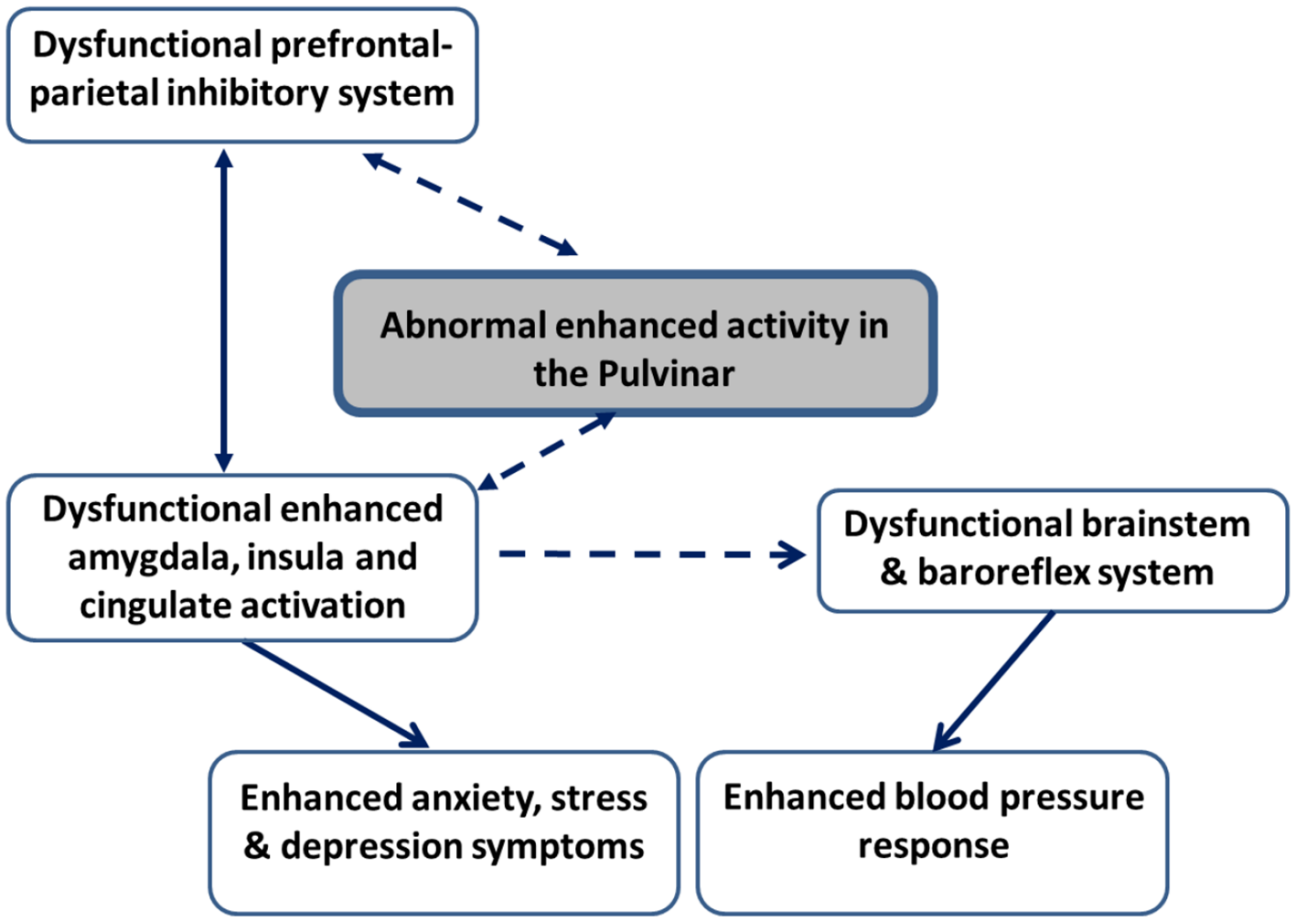
Schematic illustration of the neural pathways hypotheses in patients with essential hypertension and groups at high risk to develop hypertension. Hypothesized connectivity is represented with dashed lines. Known connectivity is represented with continuous lines. Enhanced activation in the pulvinar in face of stimuli interpreted as threatening is expected to result in enhanced activity in limbic regions, including the amygdala, insula, and cingulate regions both directly and indirectly, via the influence of the pulvinar on dysfunctional inhibitory activation in prefrontal and parietal areas. This enhanced limbic activation, in turn, is expected to lead to enhanced symptoms of anxiety, stress, and depression. In addition, enhanced limbic activation is hypothesized to result in dysfunctional brainstem and baroreflex regulatory mechanisms, which are expected to result in exaggerated blood pressure reactions to aversive stimuli.

There is evidence of abnormalities in prefrontal-limbic neural pathways among patients with EH and those at high risk (Gianaros et al., 2009, 2012). Further evidence shows that central aortic and peripheral BP measures are related to cognitive functions (Hughes and Sink, 2016; Aronow, 2017). A large sample study (*N* = 493) found that higher BP was related to impairment in several cognitive processes, among them poorer color-word Stroop processing, which is commonly used to assess the ability to inhibit cognitive interference (Pase et al., 2013). Taken together, these studies indirectly suggest that deficient neurocognitive inhibitory control mechanisms may form the basis for the abnormally enhanced emotional reactions seen in groups at high risk for developing EH. In a first attempt to examine whether inhibitory control mechanisms influence BP reactions among healthy volunteers and to determine the neural basis of this modulation, Okon-Singer et al. (2014) manipulated attention to distracting highly aversive pictures while simultaneously measuring neural activation using fMRI and peripheral BP. The results demonstrated that attention modulates BP and neural reactions to aversive stimuli in a network that includes prefrontal, parietal, limbic, and brainstem regions previously shown to be related both to emotion control and to BP reactivity. These results indicate that neurocognitive control mechanisms modulate BP reactions among healthy individuals and indirectly suggest that abnormalities in these systems may underlie abnormal BP emotional reactions (Okon-Singer et al., 2014). Based on these findings, it is plausible to hypothesize that among patients with EH, abnormalities in prefrontal and parietal areas associated with inhibitory control results in deficits in emotion regulation, which leads to enhanced activity in the amygdala, insula and cingulate cortex. This enhanced activity, in turn, leads to elevated symptoms of anxiety and depression, as well as exaggerated BP reactions to stress and aversive stimuli (see Figure 1). However, this hypothesis should be taken with caution and directly examined in future studies.

## The pulvinar may Play an Important Role in Aberrant Emotion Regulation in Essential Hypertension

Recent models highlight the role of the thalamic pulvinar nucleus in emotion regulation, specifically in the interplay between emotion and attention in early emotion regulation mechanisms (Pessoa, 2017). This view is based on two types of evidence: First, the pulvinar has substantial anatomical connections with diffused brain regions, including retinal, striatal and extrastriatal cortices, frontal, parietal, orbital and temporal cortices, the superior colliculus, and the amygdala (Grieve et al., 2000) and was recently suggested as a central node in a functional network related to emotion-cognition interactions (Pessoa, 2017). Specifically, while the pulvinar is considered to be an area of the brain irrelevant to the study of higher cognition and is therefore often disregarded (Silverstein and Ingvar, 2015), it has extensive connections to visual and fronto-parietal areas important for attention and to the amygdala, which is important for emotion (Grieve et al., 2000; Buchsbaum et al., 2006; Tamietto et al., 2012). Evidence suggests that the pulvinar may play an important role in selective orienting of visual attention to relevant stimuli (Fischer and Whitney, 2012), including selective attention to emotional/aversive stimuli (Padmala et al., 2010; Frank and Sabatinelli, 2014). Pulvinar connectivity has also been implicated in emotion processes underlying anxiety. In an effective connectivity analysis, Tadayonnejad et al. (2016) demonstrated a causal relation between the pulvinar and higher order visual and frontal areas among participants with social anxiety in an emotional face-processing paradigm. Second, there is evidence for pulvinar involvement in emotional tasks, including tasks that involve threat detection. For example, Hakamata et al. (2016) showed that individuals with attention bias to aversive information exhibited higher pulvinar activation with unattended fearful faces than with unattended neutral faces, as well as enhanced effective connectivity from the pulvinar to fronto-parietal areas. Based on data from patients with brain injuries, we (Arend et al., 2015) suggested that the pulvinar may determine whether a certain stimulus is considered to be emotional and therefore receive prioritized processing. In line with our suggestion, Hakamata et al. (2016) concluded that the pulvinar may be involved in gating unattended aversive information depending on individual threat-related attention bias. These researchers later added data to bolster these findings (Hakamata et al., 2018). The pulvinar has also been linked to stress and post-traumatic stress disorder (Drabant et al., 2012; Terpou et al., 2018). Indirect evidence further indicates that the pulvinar is related to action and BP reactions (Kemper et al., 2001; Renard et al., 2014).

Although the pulvinar is thought to play a critical and active role in EH, the underlying mechanisms and links between these findings remain unclear. Based on its anatomical and functional connectivity, we hypothesize that pulvinar may influence both BP and anxiety and depression symptoms via limbic regions (Figure 1). Specifically, pulvinar activation may lead to enhanced limbic activation, which in turn results in higher anxiety and depression behaviors, as well as exaggerated BP reactions to aversive stimuli, possibly due to abnormalities in brainstem and baroreflex mechanisms. This hypothesis should be directly examined in future studies.

## Conclusions and Outlook

In the current paper, we highlighted the gap in knowledge about factors underlying deficient emotion regulation in EH, a context that is of high clinical significance. By bringing together separate yet related strands of research, we conclude that aberrant emotion regulation in EH may share common neurocognitive mechanisms with stress and anxiety. Furthermore, we suggest that the role of the thalamic pulvinar nucleus in EH, anxiety, stress, and emotion regulation may be a promising area for investigation.

Future studies may also investigate individuals at high risk of developing EH, such as individuals with prehypertension or individuals with a genetic risk. Indeed, recent findings (Schaare et al., 2019) demonstrate lower gray matter in thalamic, amygdala, prefrontal and parietal regions in prehypertension. Furthermore, recent technological advances provide continuous non-invasive methods for measuring and analyzing BP, which can also assist in future investigations (Wiener et al., 2020). It is our hope that future studies will address these questions, so that in the long-term new treatments can be developed and help individuals with EH to more effectively combat daily life stressors and reduce their impact on physical and mental health.

## Author Contributions

AW and HO-S: initiated the idea, literature review, and writing. CR: literature review and writing. NN and AV: contribution to the conception of the work and involved in the writing.

## Conflict of Interest

The authors declare that the research was conducted in the absence of any commercial or financial relationships that could be construed as a potential conflict of interest.
